# Detection of high PD-L1 expression in oral cancers by a novel monoclonal antibody L_1_Mab-4

**DOI:** 10.1016/j.bbrep.2018.01.009

**Published:** 2018-02-06

**Authors:** Shinji Yamada, Shunsuke Itai, Mika K. Kaneko, Yukinari Kato

**Affiliations:** Department of Antibody Drug Development, Tohoku University Graduate School of Medicine, 2-1 Seiryo-machi, Aoba-ku, Sendai, Miyagi 980-8575, Japan

**Keywords:** PD-L1, Programmed cell death-ligand 1, PD-1, programmed cell death-1, CBIS, cell-based immunization and screening, SCC, squamous cell carcinoma, ACC, adenoid cystic carcinoma, MEC, mucoepidermoid carcinoma, CTLA-4, cytotoxic T-lymphocyte-associated antigen 4, HNC, head and neck cancer, APC, antigen-presenting cell, DMEM, Dulbecco's Modified Eagle's Medium, EDTA, ethylenediaminetetraacetic acid, BSA, bovine serum albumin, PBS, phosphate-buffered saline, FBS, fetal bovine serum, DAB, 3,3-diaminobenzidine tetrahydrochloride, Programmed cell death-ligand 1, Monoclonal antibody, Oral cancer

## Abstract

Programmed cell death-ligand 1 (PD-L1), which is a ligand of programmed cell death-1 (PD-1), is a type I transmembrane glycoprotein that is expressed on antigen-presenting cells and several tumor cells, including melanoma and lung cancer cells. There is a strong correlation between human PD-L1 (hPD-L1) expression on tumor cells and negative prognosis in cancer patients. In this study, we produced a novel anti-hPD-L1 monoclonal antibody (mAb), L_1_Mab-4 (IgG_2b_, kappa), using cell-based immunization and screening (CBIS) method and investigated hPD-L1 expression in oral cancers. L_1_Mab-4 reacted with oral cancer cell lines (Ca9-22, HO-1-u-1, SAS, HSC-2, HSC-3, and HSC-4) in flow cytometry and stained oral cancers in a membrane-staining pattern. L_1_Mab-4 stained 106/150 (70.7%) of oral squamous cell carcinomas, indicating the very high sensitivity of L_1_Mab-4. These results indicate that L_1_Mab-4 could be useful for investigating the function of hPD-L1 in oral cancers.

## Highlights

• Programmed cell death-ligand 1 (PD-L1) is expressed in many cancers.
• PD-L1 expression has not been fully investigated in oral cancers.
• A novel anti-PD-L1 mAb L_1_Mab-4 was developed in this study.
• L_1_Mab-4 stained 70.7% of oral SCCs in a membrane-staining pattern.

## 1 Introduction

Oral cancer is the eleventh highest of all cancer types [1] and constitutes approximately 2% of all cancer cases worldwide [2]. In oral cancers, there are certain histological tumors, including squamous cell carcinoma (SCC), adenosquamous cell carcinoma, adenoid cystic carcinoma, osteosarcoma, and mucoepidermoid carcinoma. SCC is the most common type, accounting for >90% of oral cancers [3]. Because of improvements and progression in therapeutic techniques such as surgery, chemotherapy, and radiotherapy, the 5-year survival rate has reached >80% [4], [5]. In contrast, it is sometimes difficult to provide sufficient therapeutic effects because of the risks of adverse events [6], [7], [8].

Recently, tumor immunotherapies, which is focused on several immune checkpoint molecules, such as programmed cell death-1 (PD-1), programmed cell death-ligand 1 (PD-L1), and cytotoxic T-lymphocyte-associated antigen 4 (CTLA-4), have emerged. Clinically, nivolumab, a complete human IgG_4_ against PD-1, was firstly approved for the treatment of recurrent and/or metastatic head and neck cancer, which was previously treated with platinum-based chemotherapy [9]. Avelumab, a complete human IgG_1_ against PD-L1, was recently approved for the treatment of metastatic Merkel cell carcinoma [10], [11], which is detected in oral cavity [12].

PD-L1, also known as B7-H1 and CD274, is a type I transmembrane glycoprotein, which is expressed on antigen-presenting cells and some tumor cells, including melanoma, ovarian, and lung cancer cells [13], [14], [15]. PD-L1 is a ligand for PD-1 and is involved in inhibiting T-cell effector functions [16], leading to the escape of tumor cells from immune response. Recent studies have revealed a strong correlation between PD-L1/PD-L2 expression on tumor cells and negative prognosis in cancer patients [17], [18], [19].

Lin *et al.* have revealed a correlation between high PD-L1 expression and metastasis and poor prognosis in oral SCC [20]. Several reports have shown that PD-L1 could be an effective target for treatment [21], [22], [23], [24], [25], [26]. However, PD-L1 expression in oral cancers has not been completely investigated. In this study, we established a novel anti-PD-L1 antibody and performed immunohistochemistry for oral cancers.

## 2 Materials and methods

### 2.1 Cell lines

Ca9-22, HO-1-u-1, SAS, HSC-2, HSC-3, HSC-4, and HEK-293T cells were obtained from the Japanese Collection of Research Bioresources Cell Bank (Osaka, Japan). LN229 and P3U1 cell lines were obtained from the American Type Culture Collection (ATCC, Manassas, VA). LN229/human PD-L1 (hPD-L1) was produced by transfecting pCAG/PA-hPD-L1-RAP-MAP into LN229 cells using a Gene Pulser Xcell electroporation system (Bio-Rad Laboratories, Inc., Berkeley, CA). The stable transfectant of LN229/hPD-L1 was established by limiting dilution. HEK-293T/hPD-L1 was produced by transfecting pCAG/PA-hPD-L1-RAP-MAP into HEK-293T cells using Neon transfection system (Thermo Fisher Scientific, Inc., Waltham, MA). A few days after transfection, PA tag-positive cells were sorted using a cell sorter (SH800; Sony Corp., Tokyo, Japan). PA tag system (GVAMPGAEDDVV (12 a.a.) vs. clone: NZ-1), RAP tag system (DMVNPGLEDRIE (12 a.a.) vs. clone: PMab-2), and MAP tag system GDGMVPPGIEDK (12 a.a.) vs. clone: PMab-1) have been previously established in Tohoku University Graduate School of Medicine and described in detail [27], [28], [29].

Ca9-22, HO-1-u-1, SAS, HSC-2, HSC-3, HSC-4, LN229, LN229/hPD-L1, HEK-293T, and HEK-293T/hPD-L1 cells were cultured in Dulbecco's Modified Eagle's Medium (DMEM; Nacalai Tesque, Kyoto, Japan), and P3U1 cell line was cultured in RPMI 1640 medium (Nacalai Tesque) at 37 °C in a humidified atmosphere containing 5% CO_2_ and 95% air, both of which were supplemented with 10% heat-inactivated fetal bovine serum (FBS; Thermo Fisher Scientific, Inc.). One hundred units/mL penicillin, 100 μg/mL streptomycin, and 25 μg/mL amphotericin B (Nacalai Tesque) were added to the culture medium. Zeocin (0.5 mg/mL; InvivoGen, San Diego, CA) was added to the culture medium of LN229/hPD-L1 and HEK-293T/hPD-L1.

### 2.2 Animals and human tissues

Female 4-week-old BALB/c mice were purchased from CLEA Japan (Tokyo, Japan) and kept under specific pathogen-free conditions. The Animal Care and Use Committee of Tohoku University approved all animal experiments described in this study. Oral cancer tissue arrays were purchased from US Biomax, Inc. (Rockville, MD): Cases 1–38 from Cat. # OR480, Cases 39–88 from Cat. # OR601b or from Cybrdi, Inc. (Frederick, MD), and Cases 89–159 from Cat. # 27-10-001. The study examined one patient (Case-160) with oral cancer who underwent surgery at the Tokyo Medical and Dental University. The Tokyo Medical and Dental University Institutional Review Board reviewed and approved the use of human cancer tissues, and written informed consent was obtained for using the human cancer tissue samples.

### 2.3 Hybridoma production

One BALB/c mouse was immunized using intraperitoneal (i.p.) injections of LN229/hPD-L1 (1 × 10^8^ cells) together with Imject Alum (Thermo Fisher Scientific Inc.). After three additional immunizations, a booster injection of LN229/hPD-L1 was intraperitoneally administered 2 days before harvesting the spleen cells. Spleen cells were then fused with P3U1 cells using PEG1500 (Roche Diagnostics, Indianapolis, IN). The resulting hybridomas were grown in RPMI medium supplemented with 10% FBS and hypoxanthine, aminopterin, thymidine selection medium supplement (Thermo Fisher Scientific Inc.), and 5% BriClone Hybridoma Cloning Medium (QED Bioscience Inc., San Diego, CA). One hundred units/mL penicillin, 100 μg/mL streptomycin, and 25 μg/mL amphotericin B (Nacalai Tesque) were added to the medium. Plasmocin (5 μg/mL; InvivoGen) was also used to prevent *Mycoplasma* contamination. Culture supernatants were screened by SA3800 Cell Analyzers (Sony Corp.) using LN229 and LN229/hPD-L1. MAbs were purified from the supernatants of hybridomas and cultured in Hybridoma-SFM medium (Thermo Fisher Scientific Inc.) using Protein G Sepharose 4 Fast Flow (GE Healthcare UK Ltd, Buckinghamshire, England).

### 2.4 Flow cytometry

Cells were harvested by briefly exposing to 0.25% trypsin/1-mM ethylenediaminetetraacetic acid (EDTA; Nacalai Tesque, Inc.). After washing with 0.1% bovine serum albumin (BSA)/phosphate-buffered saline, the cells were treated with 1 μg/mL of anti-PD-L1 mAbs, such as clones 29E.2A3 [30] (BioLegend, Inc., San Diego, CA) and L_1_Mab-4 (produced in this study), for 30 min at 4 °C and subsequently with Alexa Fluor 488-conjugated anti-mouse IgG (1:1000; Cell Signaling Technology, Inc., Danvers, MA). Fluorescence data were collected using EC800 Cell Analyzers (Sony Corp.).

### 2.5 Immunohistochemical analyses

Histological sections of 4-μm thickness were deparaffinized in xylene and subsequently rehydrated and autoclaved in citrate buffer (pH 6.0; Nichirei Biosciences, Inc., Tokyo, Japan) or EnVision FLEX Target Retrieval Solution, High pH (Agilent Technologies Inc., Santa Clara, CA) for 20 min. Sections were then incubated with 10 μg/mL of L_1_Mab-4 for 1 h at room temperature, treated using an Envision+ kit (Agilent Technologies Inc.) for 30 min. Color was developed using 3,3-diaminobenzidine tetrahydrochloride (DAB; Agilent Technologies Inc.) for 2 min, and counterstained with hematoxylin (Wako Pure Chemical Industries Ltd., Osaka, Japan).

## 3 Results and discussion

### 3.1 Production of novel anti-hPD-L1 mAbs using cell-based immunization and screening (CBIS) method

**Fig. 1:**
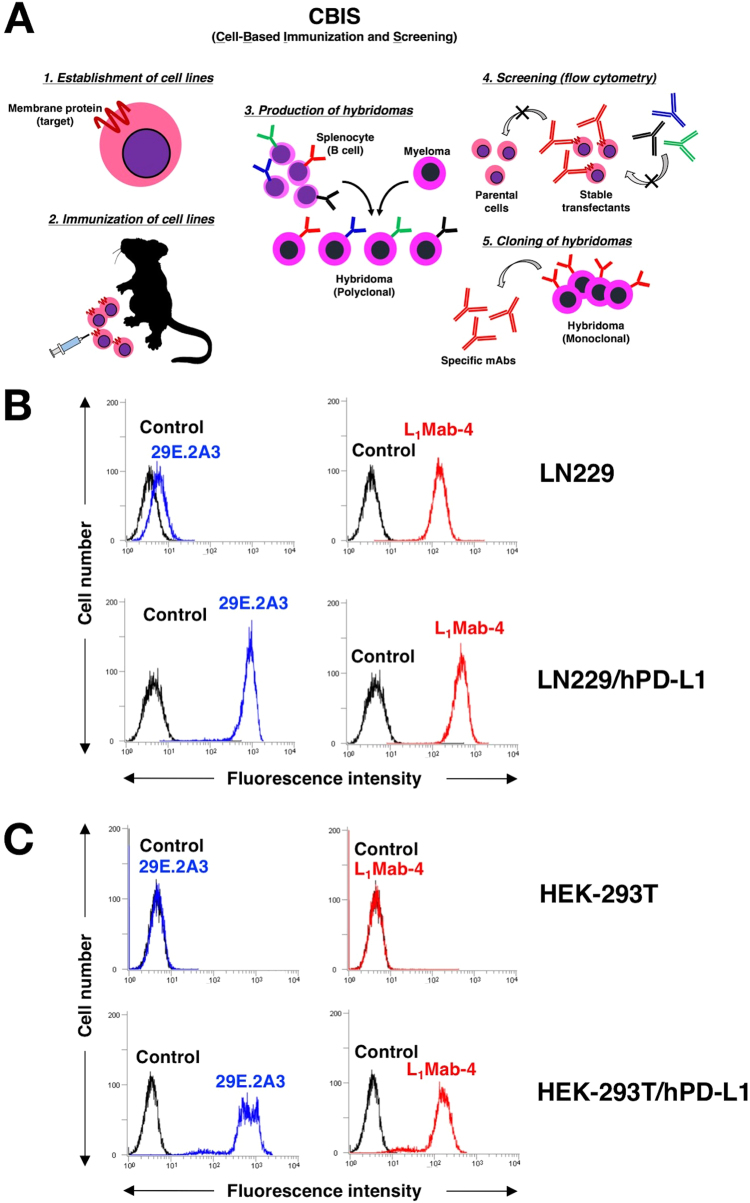
**Flow cytometry of L**_**1**_**Mab-4 against LN229/hPD-L1.** (A) Procedure of cell-based immunization and screening (CBIS) methods. (B) LN229 and LN229/hPD-L1 were treated with 1 μg/mL of 29E.2A3 (positive control; blue line) and L_1_Mab-4 (red line), followed by treatment with Alexa Fluor 488-conjugated anti-mouse IgG; black line, negative control. (C) HEK-293T and HEK-293T/hPD-L1 were treated with 1 μg/mL of 29E.2A3 (positive control; blue line) and L_1_Mab-4 (red line), followed by treatment with Alexa Fluor 488-conjugated anti-mouse IgG; black line, negative control. (For interpretation of the references to color in this figure legend, the reader is referred to the web version of this article.)

We immunized one mouse with LN229/hPD-L1 cells and performed flow cytometric screening. We previously named this cell-based strategy as the CBIS method, as shown in Fig. 1A [31], [32]. Culture supernatants of 672 wells were mixed with LN229/hPD-L1 and LN229 cells, and 27 wells (27/672; 4.0%), showing a stronger reaction against LN229/hPD-L1 cells than LN229 cells, were selected. After limiting dilution, 10 clones were established, of which seven were classified as IgG_1_ subclass and three as IgG_2b_ subclass. Only one clone, L_1_Mab-4 (IgG_2b_, kappa), reacted with lung SCC tissue, which was previously diagnosed as PD-L1-positive in an immunohistochemical analysis (data not shown). Although both L_1_Mab-4 and 29E.2A3 (mouse IgG_2b_, kappa; positive control) [30] reacted with LN229 and LN229/hPD-L1, the reactivity of L_1_Mab-4 was higher for LN229/hPD-L1 than LN229 (Fig. 1B). Both L_1_Mab-4 and 29E.2A3 also reacted with HEK-293T/PD-L1, but not with HEK-293T (Fig. 1C), indicating the specificity of L_1_Mab-4 against hPD-L1.

### 3.2 Flow cytometric analysis against oral cancer cell lines

**Fig. 2:**
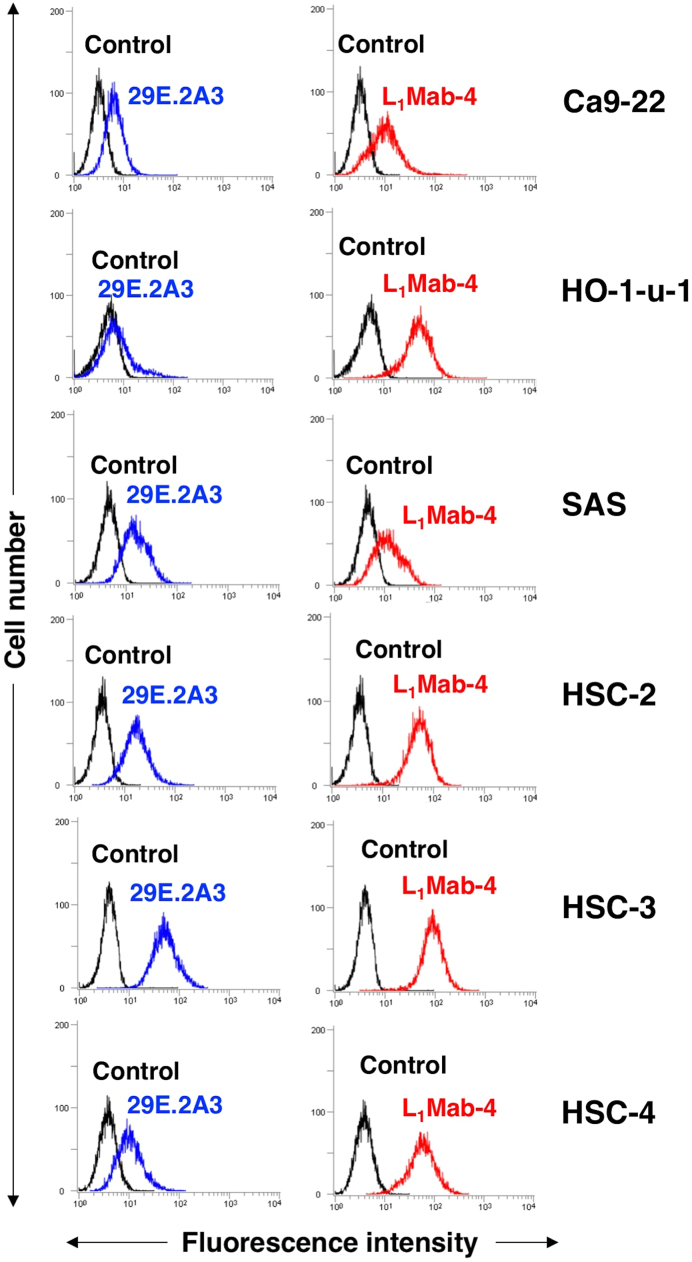
**Flow cytometry of L**_**1**_**Mab-4 against oral cancer cell lines.** Cells were treated with 1 μg/mL of 29E.2A3 (positive control; blue line) and L_1_Mab-4 (red line), followed by treatment with Alexa Fluor 488-conjugated anti-mouse IgG; black line, negative control. (For interpretation of the references to color in this figure legend, the reader is referred to the web version of this article.)

L_1_Mab-4 recognized endogenous hPD-L1 in oral cancer cell lines, such as Ca9-22, HO-1-u-1, SAS, HSC-2, HSC-3, and HSC-4 (Fig. 2). Although 29E.2A3 also reacted with these cell lines, the reaction of L_1_Mab-4 was higher than that of 29E.2A3, indicating the usefulness of L_1_Mab-4 for the detection of hPD-L1 in flow cytometry.

### 3.3 Immunohistochemical analysis against oral cancer tissues

**Fig. 3:**
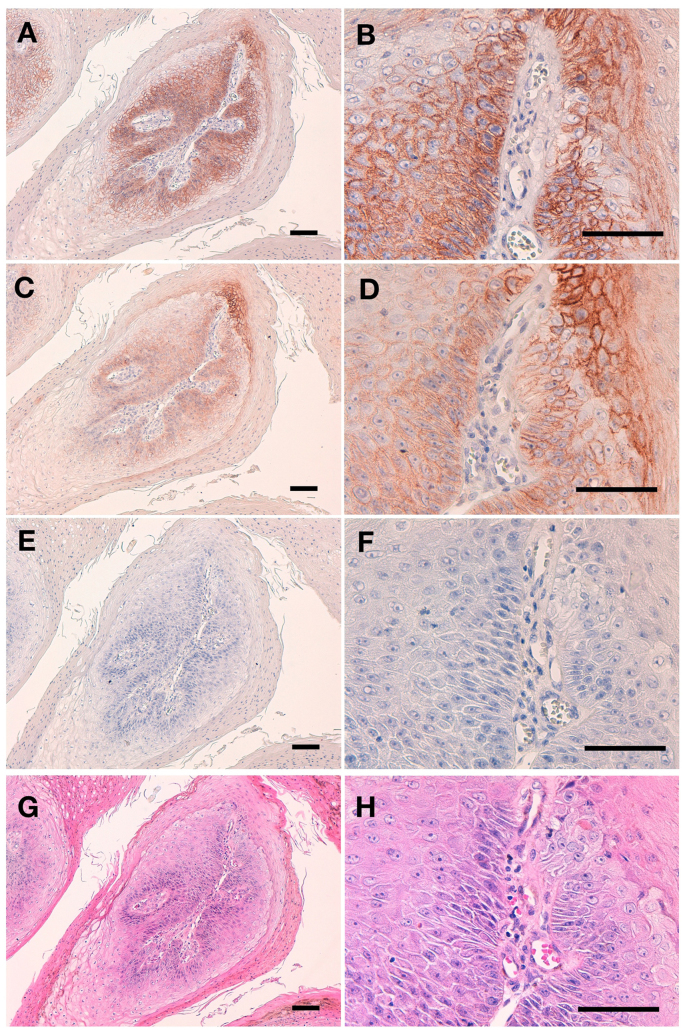
**Immunohistochemical analysis of L**_**1**_**Mab-4 against oral SCCs.** After antigen retrieval, sections were incubated with 10 μg/mL of L_1_Mab-4 followed by treatment with Envision+ kitn. Color was developed using 3,3-diaminobenzidine tetrahydrochloride (DAB), and sections were counterstained with hematoxylin. (**A, B**) Antigen retrieval using citrate buffer, pH 6; (**C, D**) Antigen retrieval using EnVision FLEX Target Retrieval Solution High pH; (**E, F**) Control; (**G, H**) Hematoxylin & eosin (HE) staining. Scale bar = 100 µm.

We further investigated the immunohistochemical utility of L_1_Mab-4 in human oral cancers. First, we performed immunohistochemical analysis using two different conditions of antigen retrieval. Fig. 3 shows that antigen retrieval using citrate buffer (pH 6) revealed a better staining pattern (Fig. 3A and B) than did EnVision FLEX Target Retrieval Solution, High pH (Fig. 3C and D). Therefore, we employed citrate buffer (pH 6) for antigen retrieval in this study. L_1_Mab-4 stained oral cancer cells in a membrane-staining pattern, indicating that L_1_Mab-4 is very useful for immunohistochemical analysis against oral cancers.

**Fig. 4:**
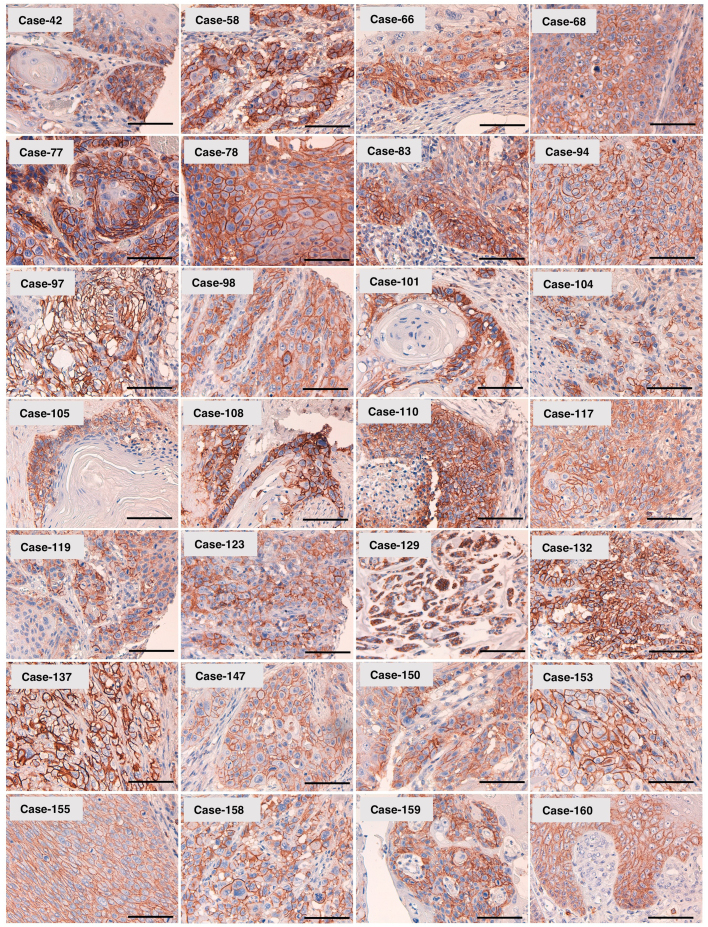
**Immunohistochemical analysis of L**_**1**_**Mab-4 against oral cancers (tissue microarray).** After antigen retrieval, sections were incubated with 10 μg/mL of L_1_Mab-4 followed by treatment with Envision+ kit. Color was developed using 3,3-diaminobenzidine tetrahydrochloride (DAB), and sections were counterstained with hematoxylin. Scale bar = 100 µm.

We then stained oral cancer tissue arrays, and the typical results (staining level, 3+) are shown in Fig. 4. L_1_Mab-4 strongly stained the cell membranes of oral cancers. As shown in Supplementary Tables 1 and 2, 106/150 (70.7%) of SCCs were stained by L_1_Mab-4 and 26/106 (24.5%) were diagnosed as 3+. Although the samples were limited, adenoid cystic carcinomas (ACCs) and mucoepidermoid carcinomas (MECs) also showed positive staining by L_1_Mab-4.

In conclusion, we successfully produced L_1_Mab-4, a novel anti-hPD-L1 mAb, using the CBIS method. This method is very advantageous because it does not require the membrane protein to be purified in all steps of mAb production. L_1_Mab-4 is very useful in the detection of hPD-L1 using flow cytometry and immunohistochemical analysis. In the near future, studies investigating the reactivity of L_1_Mab-4 for other human cancers need to be conducted. Furthermore, we should consider the possibility that L_1_Mab-4 might cross-react with other membrane proteins or react with post-translational modification, including glycosylation, on PD-L1 because the reaction of L_1_Mab-4 is much higher than those of 29E.2A3 against LN229 and HO-1-u-1 (Fig. 1, Fig. 2).
