# Supplementary material for: Detection of high PD-L1 expression in oral cancers by a novel monoclonal antibody L1Mab-4
Source: Biochem Biophys Rep. 2018 Feb 6;13:123–8. doi: 10.1016/j.bbrep.2018.01.009 (PMC5857169; doi:10.1016/j.bbrep.2018.01.009)
Supplement: Supplementary file 2 — Supplementary material [file mmc1.docx]

**Conflict of Interest**

The authors declare no conflicts of interest involving this article.
