# Supplementary material for: Detection of high PD-L1 expression in oral cancers by a novel monoclonal antibody L1Mab-4
Source: Biochem Biophys Rep. 2018 Feb 6;13:123–8. doi: 10.1016/j.bbrep.2018.01.009 (PMC5857169; doi:10.1016/j.bbrep.2018.01.009)
Supplement: Supplementary file 3 — Supplementary material [file mmc2.docx]

Supplementary Table 1. Summary of immunostaining using L_1_Mab-4.

| Tumor type | No. of cases | L1Mab-4 immunostaining | | | | No. of positive cases |
| --- | --- | --- | --- | --- | --- | --- |
|  |  | - | 1+ | 2+ | 3+ |  |
| SCC | 150 | 44 | 16 | 64 | 26 | 106/150 (70.7%) |
| ACC | 7 | 6 | 0 | 0 | 1 | 1/7 (14.3%) |
| MEC | 3 | 2 | 0 | 0 | 1 | 1/3 (33.3%) |
| Total | 160 | 52 | 16 | 64 | 28 | 108/160 (67.5%) |

SCC; squamous cell carcinoma, ACC; adenocystic carcinoma
MEC; mucoepidermoid carcinoma

The intensity of staining was evaluated as -, 1+, 2+, 3+.
